# Collagen Glycation Detected by Its Intrinsic Fluorescence

**DOI:** 10.1021/acs.jpcb.1c05001

**Published:** 2021-09-24

**Authors:** Rhona Muir, Shareen Forbes, David J.S. Birch, Vladislav Vyshemirsky, Olaf J. Rolinski

**Affiliations:** † Photophysics Group, Department of Physics, University of Strathclyde, Scottish Universities Physics Alliance, Glasgow G4 0NG, U.K.; ‡ BHF Centre for Cardiovascular Science, The Queen’s Medical Research Institute, University of Edinburgh, Edinburgh EH16 4TJ, U.K.; § School of Mathematics and Statistics, University of Glasgow, Glasgow G12 8QQ, U.K.

## Abstract

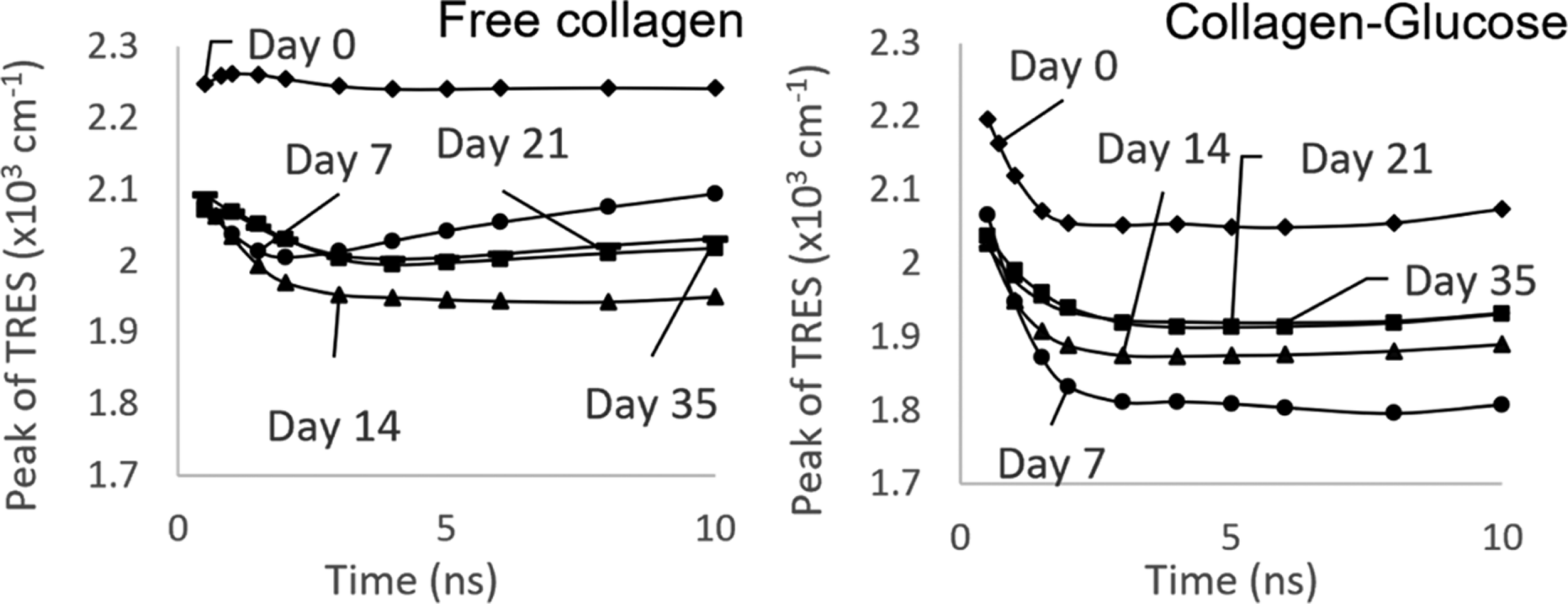

Collagen’s long half-life (in skin approximately 10 years) makes this protein highly susceptible to glycation and formation of the advanced glycation end products (AGEs). Accumulation of cross-linking AGEs in the skin collagen has several detrimental effects; thus, the opportunity for non-invasive monitoring of skin glycation is essential, especially for diabetic patients. In this paper, we report using the time-resolved intrinsic fluorescence of collagen as a biomarker of its glycation. Contrary to the traditional fluorescence intensity decay measurement at the arbitrarily selected excitation and detection wavelengths, we conducted systematic wavelength- and time-resolved measurements to achieve time-resolved emission spectra. Changes in the intrinsic fluorescence kinetics, caused by both collagen aggregation and glycation, have been detected.

## Introduction

Diabetes is a chronic, progressive disease characterized by elevated blood glucose levels. The increased amount of glucose in the blood associated with diabetes leads to various diabetic complications^1^ such as cardiovascular and vascular diseases, stroke, kidney failure, amputation, retinopathy, and neuropathy.^2,3^ It has been reported^4^ that these complications are frequently related to the formation of compounds known as advanced glycation end products (AGEs).^5,6^

Proteins with a long biological half-life are more likely than other proteins to experience glycation and AGE formation. Collagen half-life varies between tissues, and in skin is approximately 10 years,^5^ which makes it highly susceptible to glycation and AGE formation.^6^ On collagen, glucose binds to a free lysine residue^7^ to form a Schiff Base and follows the classical glycation pathway. The AGEs can be in the form of adducts or cross-links within or between the proteins.^8^ Accumulation of cross-linking AGEs in collagen has several detrimental effects: the collagen triple helix becomes less soluble and flexible,^5^ reduced arterial and myocardial compliance, increased vascular stiffness, and increased diastolic function and systolic hypertension.^9^ Collagen glycation also augments formation and migration of myofibroblasts, which contributes to fibrosis in diabetes,^10^ and it is related to the duration and severity of hyperglycaemia.^11,12^

The risk of diabetes complications can be predicted by identifying the presence of AGEs in the dermal tissue. The studies have found^13^ that long-term treatment of hyperglycaemia reduced the levels of AGEs in the skin collagen. This suggests that skin AGEs, including collagen AGEs, can be used as a biomarker for diabetes complications.^14^

In this paper, we study the potential for using the time-resolved intrinsic fluorescence of collagen as a biomarker of its glycation. Fluorescence spectroscopy has been used extensively to study the formation and accumulation of AGEs both in vitro^15,16^ and in vivo.^14,17^ Specifically, the use of three naturally fluorescent amino acids: phenylalanine, tryptophan, and tyrosine,^18^ allows the non-invasive study of biological structures and processes without the addition of any extrinsic fluorophore.

Collagen’s fluorescence in the human skin^17^ originates from two sources: tyrosine (Tyr)^19,20^ and collagen cross-links. As there are no tryptophan^21^ or phenylalanine^22^ residues in collagen, they do not interfere with Tyr. It can be excited at approximately 275–280 nm and has peak fluorescence emission at 305 nm.^20^

There are two types of collagen cross-links:^23^ pepsin-digestible and collagenase-digestible. The former are excited at approximately 340 nm and emit at 400 nm, and the latter are excited at 360 nm with a fluorescence emission peak at 440 nm.^17^ Although it is not clear what structural differences there are between these two sets of cross-links, it has been confirmed experimentally^23^ that fluorescence from collagen cross-links does have two distinct bands. Kollias et al.^17^ showed that the fluorescence intensity at 400 nm greatly reduced following pepsin digestion, but this had no effect on the fluorescence emission peak at 440 nm. The opposite was true for collagenase digestion, where there was no effect on the 400 nm peak, but the emission at 440 nm almost disappeared.

AGE accumulation drives more cross-linking within collagen fibers, and so impacts its intrinsic fluorescence,^24^ making fluorescence emission spectra a valuable tool for monitoring AGE accumulation.^25,26^

Indeed, previous studies have used skin auto-fluorescence as a means of studying glycation-induced changes both in diabetic patients and healthy controls.^25,26^ The steady-state technique, however, is not able to fully explain the mechanisms of glycation and so help in the search for anti-glycation factors that could prevent AGE formation.

Here, we propose a more comprehensive detection of the intrinsic fluorescence by performing systematic time- and wavelength-resolved measurements, instead of performing a single measurement of the fluorescence intensity decay at the arbitrary selected excitation and detection wavelengths. Our method explores the time-resolved emission spectra (TRES) obtained at discrete times throughout the fluorescence decays.^27^ Changes in TRES of the intrinsic fluorophores observed in aggregating proteins are usually complex,^28,29^ as they are caused by the compilation of multiple effects, mainly the heterogeneity of the sample and the dielectric relaxation of fluorophores. In these studies, we aim to verify whether the alterations in TRES triggered by collagen glycation are sufficient to help in understanding the actual mechanisms of glycation, and thus potential pathways of its prevention.

## Materials and Methods

We have studied the time-resolved responses of tyrosine (excitation at 280 nm) and of pepsin-digestible cross-links (excitation at 340 nm) in the collagen. Two samples were analyzed: free collagen and collagen-glucose solutions.

### Tyrosine

Sample preparation followed the same protocol in both cases. Collagen type 1 solution from rat-tail and phosphate-buffered saline pH 7.4 (PBS) were purchased from Sigma-Aldrich and used to prepare a free collagen sample of concentration 10 μM. The collagen-glucose sample was then prepared by adding the glucose powder (Sigma-Aldrich) to make a sample containing 10 μM collagen and 20 mM glucose. Both samples were stored in an oven at 37 °C for the duration of the experiments. All measurements were carried out using 4 × 1 × 1 cm quartz cuvettes.

### Pepsin Digestible Cross-Links

The collagen sample had a concentration of 20 μM, and the collagen–glucose sample contained 20 μM collagen and 40 mM glucose. Higher concentrations of both collagen and glucose were used to optimize the experiment, as the experiments at the original lower concentration showed that the fluorescence intensity at 340 nm excitation wavelength was too low, compromising the proper signal-to-noise ratio.

In the glucose-containing samples, the glycated collagen was not purified from the non-glycated one. The first fluorescence measurements were taken within ∼20 min of glucose being added to the sample.

Corrected fluorescence emission spectra were obtained on a Fluorolog (HORIBA Scientific) using a resolution of 1 nm, with both the excitation and emission monochromators set to a slit width of 5 nm. The fluorescence intensity decay measurements, and therefore, the TRES were obtained using a DeltaFlex fluorescence lifetime system (Horiba Jobin Yvon IBH Ltd, Glasgow), which uses time-correlated single photon counting (TCSPC) to record fluorescence decay. Two nanoLEDs with a repetition rate of 1 MHz and the peak excitation wavelengths at 280 nm (pulse duration <1 ns) and 340 nm (pulse duration <1 ns) were used to study tyrosine and pepsin-digestible cross-links, respectively. To determine TRES, the fluorescence decays were recorded at a range of detection wavelengths (300–400 nm for Tyr, 380–500 nm for cross-links), in increments of 10 nm.

For both sub-experiments, the decays measured at each wavelength λ were then fitted to the experimental fluorescence curve *I*_exp_(t)
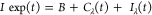
1which was assumed to be a combination of the fluorescence intensity decay *I*_λ_(*t*) represented by 2
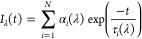
2(where α_*i*_(λ) and τ_*i*_(λ) are the *i*-*th* pre-exponential component and its related fluorescence lifetime, respectively), *B* which is the background fluorescence, and *C*_λ_(*t*) which is the scattered excitation light. Including the actual scattered excitation light *C*_λ_(*t*) (the fraction of the prompt function shifted in time due to the detecting photomultiplier’s temporal dependence on wavelength) into the model of the experimental curve enables an accurate fluorescence decay fitting also in the case when the experimental decay has a large contribution of the scattered light.^30^ The Supporting Information presents the examples of fitting eq 1 and the traditional model decay (eq 2 only, with an extra exponential component with the fixed time constant equal to 1/2 of the MCA time calibration value representing the scattered light) to the same decay data (see Figures S1–S6). The differences in the results obtained in both approaches are mostly negligible. For TRES calculations, we used the results recovered from fitting eq 1 to the data, as the model of the measured signal assumed in this approach is more realistic.

For the data obtained for both collagen and collagen-glucose samples, a 3-exponential model (*N* = 3) was found to be sufficient to describe all fluorescence decays on the basis of the goodness-of-fit criterion (χ^2^) and the distributions of residuals (see examples in the Supporting Information). Contrary to the common approach, however, we do not claim that this indicates 3-exponential kinetics but rather that the 3-exponential function is a good mathematical representation of the observed decays.

Once values for α_*i*_(λ) and τ_*i*_(λ) were obtained, they were used to construct the TRES
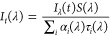
3where *S*(λ) is the steady-state fluorescence intensity at the emission wavelength λ at which fluorescence decay was recorded. The TRES was finally converted to a function of wavenumber ν
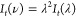
4

This procedure generates a series of fluorescence intensity spectra as a function of wavenumber ν, with each spectra representing fluorescence at a different time *t* after excitation.

The next stage involved fitting a “technical” model of TRES to the experimental TRES, namely, we used the modified multiple Toptygin-type distribution^31^ to represent the shape of spectral components.
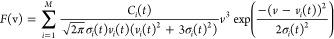
5

In this model, *M* is the number of spectral components, *C*_*i*_ is the contribution of each component to fluorescence, *v*_*i*_ is the peak position, and σ_*i*_ is the half-width of the distribution. The  term is the normalization factor for this equation. Note, that the Franck–Condon factor envelope^31^ is approximated here by a Gaussian function instead of a more realistic skewed function. This allows a reduction in the number of model 5 parameters, which was necessary because of the limited number of experimental TRES points calculated in TCSPC analysis. The model function 5 was fitted to the experimental TRES points, and the potential mechanisms of the observed changes in TRES were discussed on the basis of the goodness of fit obtained for different *M*, and the changes in *v*_*i*_(*t*), *C*_*i*_(*t*), and σ_*i*_(*t*) values. The experimental TRES points and the corresponding model TRES curves were found for day 0, and for various subsequent days after preparation. The examples of the obtained plots are shown in the Supporting Information (Figures S7 and S8).

## Results and Discussion

### Tyrosine Responses (Excitation 280 nm)

**Figure 1:**
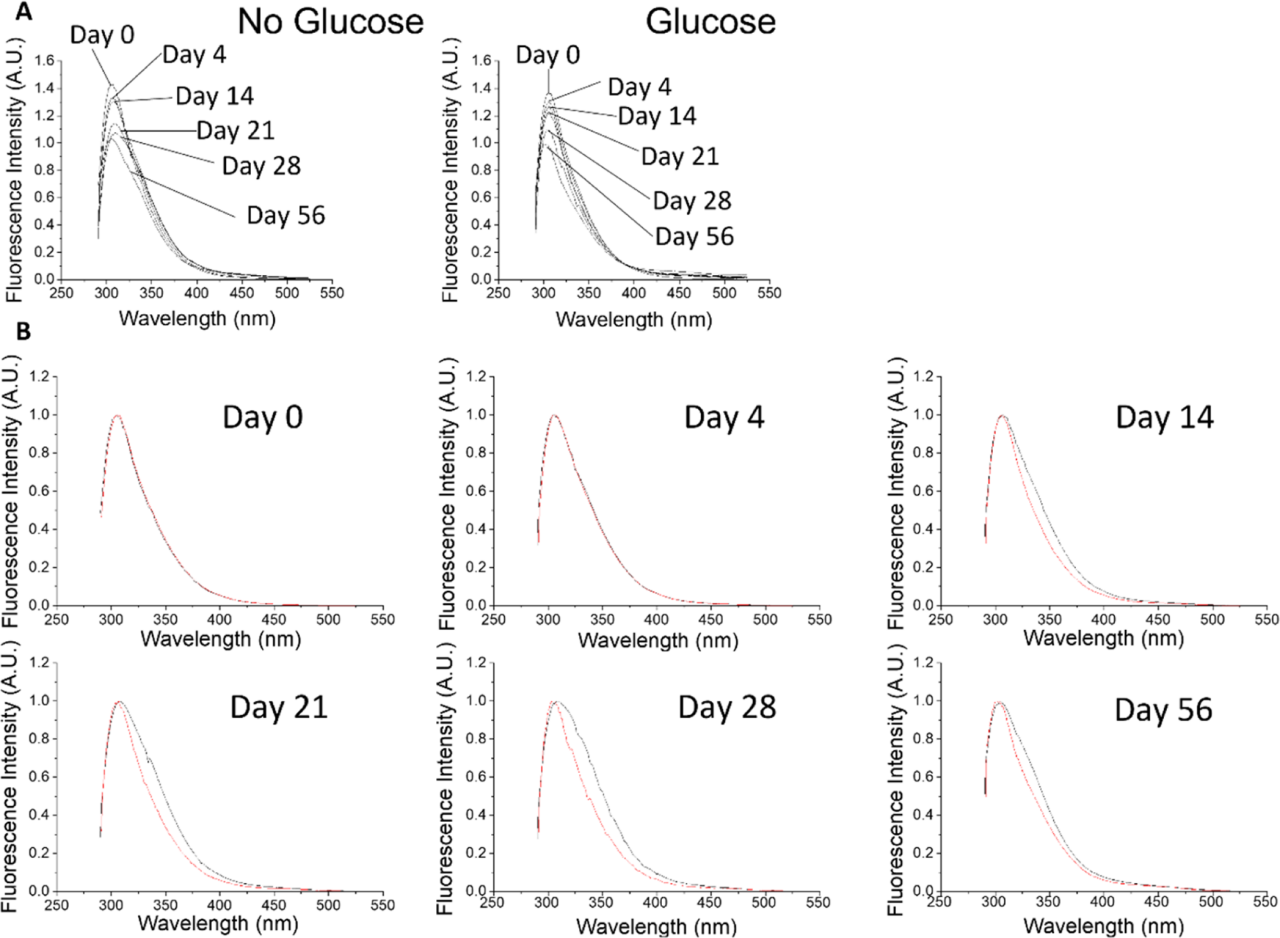
Fluorescence emission spectra for free collagen and collagen–glucose when excited at 280 nm. (A) Fluorescence changes over time for collagen and collagen–glucose at days 0, 4, 14, 21, 28, and 56. (B) Collagen (black) and collagen-glucose (red) on days 0, 4, 14, 21, 28, and 56, with both samples’ spectra normalized to their peak fluorescence.

Figure 1 shows the fluorescence emission spectra for free collagen and collagen-glucose over 56 days, where the samples were excited at 280 nm to observe the response of tyrosine. The absolute fluorescence intensities of both collagen and collagen–glucose decrease over the 56 days, as shown in Figure 1A.

Changes observed in both samples could be due to aggregation and/or glycation, as both processes may affect the conformation of the protein, including the local microenvironment of tyrosine. Figure 1B presents the pairs of spectra at individual days normalized to 1. The narrower spectra observed in the collagen–glucose sample may be a result of glycation, where the presence of the collagen–glucose aggregates reduces the formation of bigger collagen aggregates and consequently reduces the broadening of the spectrum reflecting the narrower range of the π electron spatial distribution. This hypothesis is further supported by TRES results discussed in the later section. It should be noted that we do not consider the potential degrading effects of UV illumination on the sample, as the intensities of light used in our measurements are minimal.

**Figure 2:**
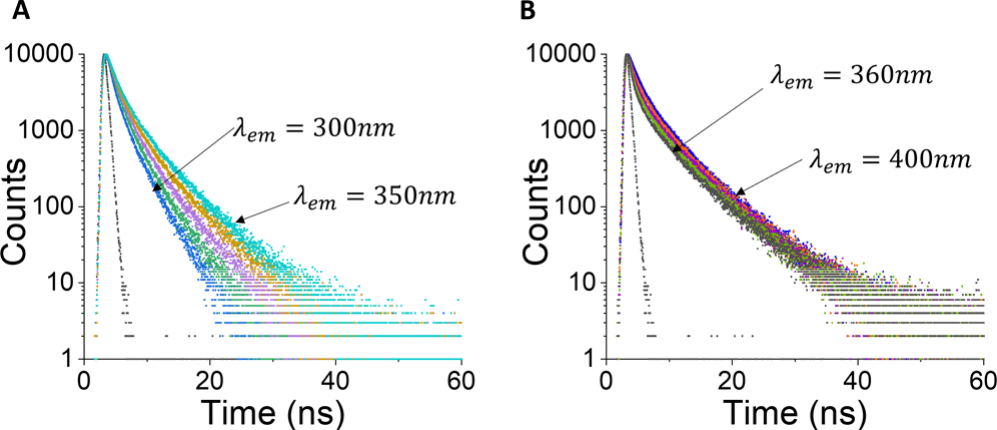
Fluorescence intensity decays for the collagen on day 0, excited at 279 nm. Part A shows the emission wavelengths from 300–350 nm in increments of 10 nm and part B shows the emission wavelengths from 360–400 nm. Both plots are shown with the prompt signal (black).

Additional information has been extracted from the analysis of TRES of both samples. This was implemented through a series of fluorescence intensity decays recorded when exciting the collagen’s Tyr at 280 nm. The decays were detected at the range 300—400 nm in increments of 10 nm (as in the example for day 0 shown in Figure 2). Figure 2 demonstrates a strong lifetime-wavelength dependence, where fluorescence at the shorter detection wavelengths decays more rapidly than at the longer wavelengths. This is consistent with the lifetime-wavelength correlation observed in proteins^32^ and is usually caused by dielectric relaxation of intrinsic fluorophores.

**Figure 3:**
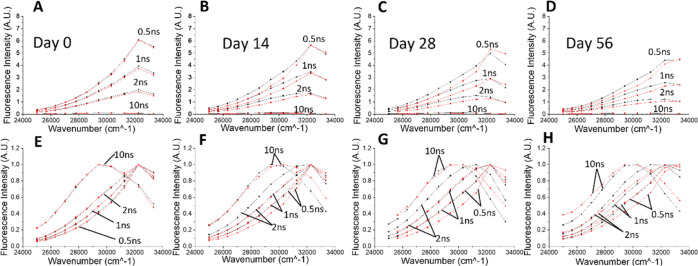
TRES spectra of free collagen (black) and collagen-glucose (red) on day 0 (A), day 14 (B), day 28 (C), and day 56 (D). Plots (E–H) show the same spectra after normalization.

The decays recorded at various times after sample preparation were used to calculate the TRES for both collagen and collagen–glucose samples. These plots, along with their related normalization plots, are shown in Figure 3. Part A (absolute spectra) and E (normalized spectra) show the result obtained for both samples on day 0, where no effect of glycation is expected yet. In this case, the spectra for both the free collagen and collagen–glucose samples are very similar, with the intensity gradually decreasing within nanoseconds. This similarity demonstrates the robustness of the experimental and data processing procedures that were used for TRES calculation.

**Figure 4:**
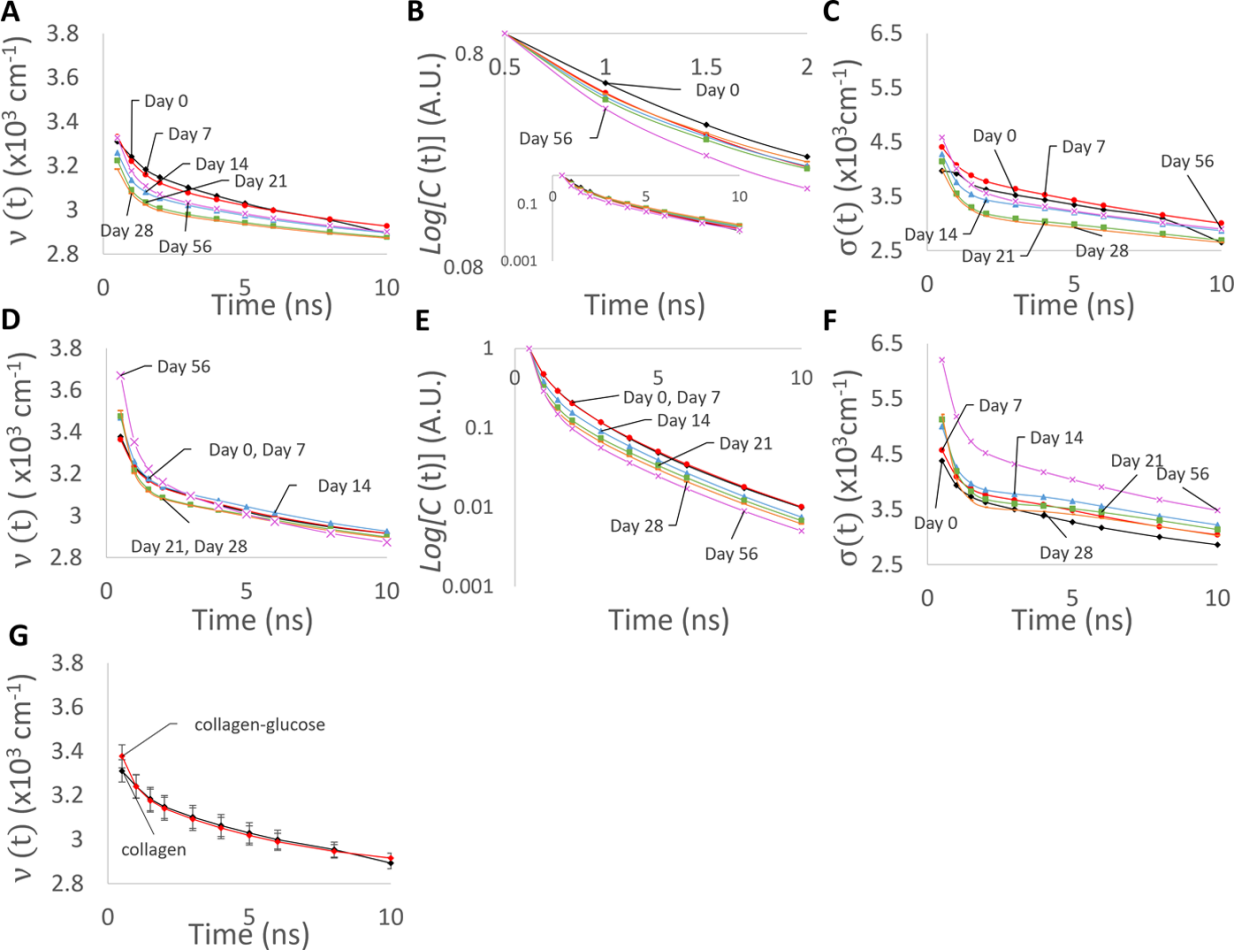
Plots (A,D) show the time evolution of the peak position *v*(*t*) of the emitting fluorophores for collagen and collagen–glucose, respectively, excited at 280 nm. The time evolution of fluorescence intensity C(*t*) is shown for collagen and collagen–glucose in plots (B,E), with each spectra normalized. The inset shows the same data over a longer time range. The six lines show day 0, day 7, day 14, day 21, day 28, and day 56. Plots (C,F) show the changes in σ parameters (see eq 5). Part (G) shows (*t*) for collagen and collagen-glucose on day 0 for comparison, with the error bars showing the 90% confidence intervals for the fitted values.

At the later times after sample preparation, differences in the TRES of both samples are observed, indicating changes in the evolution of collagen spectra caused by the presence of glucose. To obtain more quantitative information on the detected effects, the technical model of TRES (eq 5) was fitted to experimental TRES data. We used the Akaike information criterion^33^ (for definition see the Supporting Information) to optimize the number *M* of the components of the eq 5. It has been found (see Table T1 in the Supporting Information) that to avoid over parametrization of the model, only one component (*M* = 1) should be used. Note that such a result does not imply homogeneous dielectric relaxation because the same shapes of the normalized TRES are not observed here. The evolution of the recovered *v*(*t*), log(*C*(*t*)), and σ(*t*) values are plotted in Figure 4.

Initially, at day 0, the peak position of the emitting fluorophore *v*(*t*) is ∼31,500 cm^–1^ for both samples. Over the 10 ns following excitation, the peaks show a shift to longer wavelengths, ending at ∼29,200 cm^–1^. We note that at day 0 there was no difference between the spectral shifts in the free collagen and glucose-collagen samples (see Figure 4G). Over the 56 days, however, the initial peak position ν_peak_(*t*) of the un-glycated sample (Figure 4A) steadily decreased, still following the same pattern of a spectral shift toward longer wavelengths, thus suggesting collagen aggregation. On the other hand, the ν(t) in the glycated sample (Figure 4D) remained similar over the 56 days. This indicates that the aggregation of collagen is hindered by the glycation process. This observation is consistent with the narrower steady-state fluorescence spectra shown in Figure 1B. Moreover, Figure 4B,E shows that the normalized fluorescence decays *C*(*t*) of tyrosine in collagen and collagen–glucose samples, do not change substantially. Figure 4C,F presents falls in σ(*t*) factors occurring on the ns time scale, indicating that the spectrum becomes narrower at the later times after excitation. We explain this observation by the existence of the collagen aggregates characterized by a continuous and narrow distribution of spectral peaks and dielectric relaxation times, rather than two or more distinct aggregates, which would lead to the optimal *M* = 2 or more in data analysis.

To conclude this section, the broader Tyr steady-state fluorescence spectra and the systematic red shift of the TRES in the sample of free collagen suggest collagen aggregation, which leads to increasing volumes of the individual aggregates. Moreover, the narrower steady-state spectra and reduced changes in the spectral shifts observed in the presence of glycation indicates that the collagen aggregation is limited by glucose. However, glucose does not affect the fluorescence decays of tyrosines.

### Pepsin-Digestible Cross-Link Responses (Excitation 340 nm)

**Figure 5:**
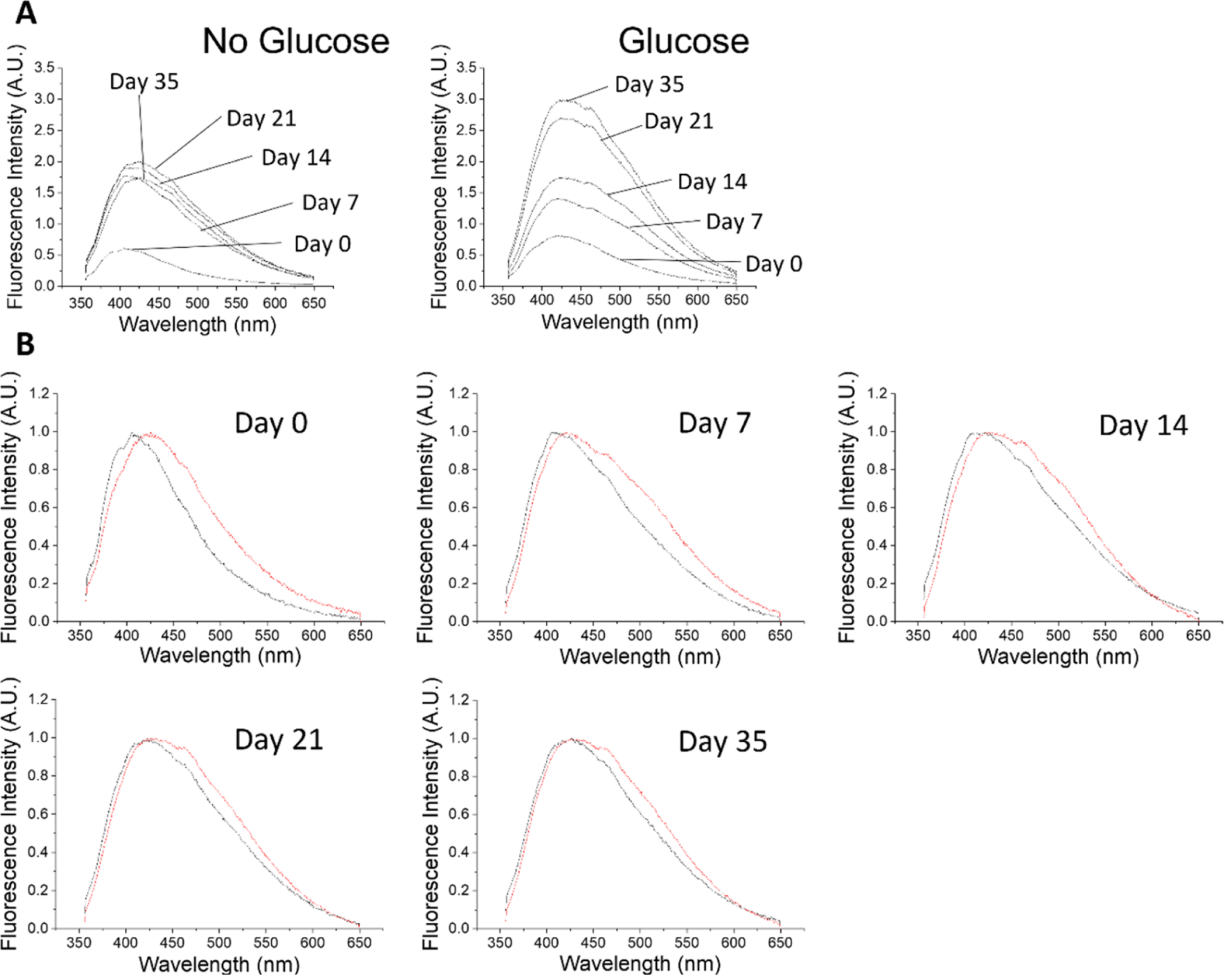
Fluorescence emission spectra for free collagen and collagen glucose when excited at 340 nm. (A) Fluorescence changes over time for collagen and collagen–glucose at days 0, 7, 14, 21, and 35. (B) Collagen (black) and collagen–glucose (red) on days 0, 7, 14, 21, and 35, with both samples spectra normalized to a peak fluorescence.

The fluorescence responses of pepsin-digestible cross-links in collagen were studied by exciting the collagen and collagen–glucose samples at 340 nm. Figure 5A shows how the steady-state fluorescence spectrum of each sample evolves over time. Free collagen initially has the fluorescence peak at ∼405 nm. The intensity of the spectrum then increases, mostly during the first 7 days, and undergoes a red spectral shift up to ∼425 nm on day 35.

The fluorescence spectrum of the collagen–glucose sample increases steadily over the 35 days. It reaches a higher value and is broader than the spectrum of the free collagen sample.

Figure 5B allows for the comparison of the fluorescence emission of free collagen and collagen–glucose samples over 35 days, with both spectra normalized. From day 0, the sample with glucose shows a broader spectrum, and there is also a difference in the wavelength of peak fluorescence: ∼405 nm for free collagen, which moves gradually to 425, and ∼425 nm for collagen–glucose, which remains stable. We attribute the emission in the 400–430 nm range to the formation of dityrosine from the neighboring tyrosine residues^34^ and this process occurs in both the free collagen and glucose–collagen samples. However, the further increase in the fluorescence intensity and the shift of the spectra observed in the collagen–glucose sample only is likely to be an indication of multiple cross-links formed between glycated collagen molecules.^14,16^

**Figure 6:**
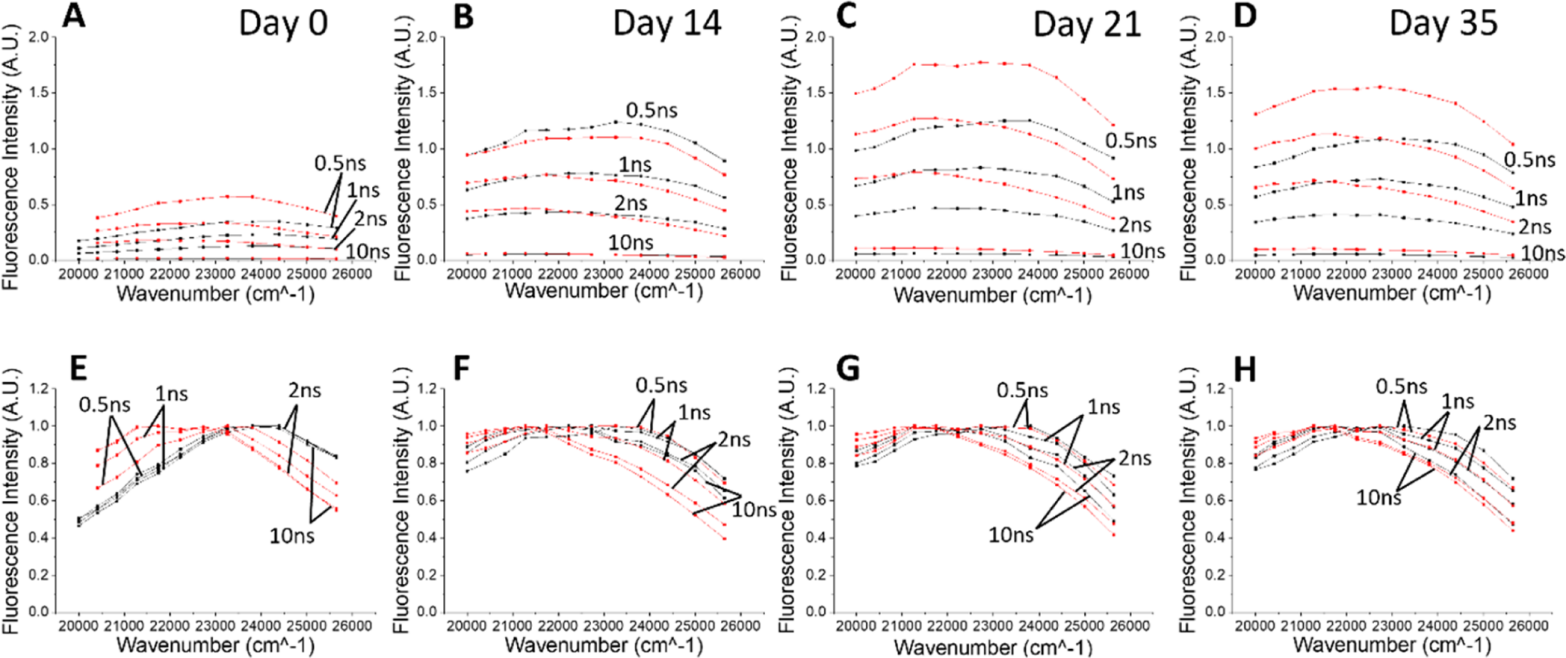
Examples of TRES spectra of free collagen (black) and collagen–glucose (red) on day 0 (A), day 14 (B), day 21 (C), and day 35 (D). Plots (E–H) show the same spectra after normalization.

To gain further information, TRES measurements were performed at various time points after sample preparation, at detection wavelengths from 390–500 nm. The absolute and normalized TRES for collagen and collagen–glucose are presented in Figure 6.

The TRES of free collagen (black curves) on day 0 show no change in their shape during the decay (see the plot of normalized spectra, black curves in Figure 6E), indicating no ns time scale processes. The broad peak of the spectrum at the range ∼24,700 and ∼23,300 cm^–1^, that is, 405–429 nm, is consistent with the steady-state spectra. The measurements performed at later days show a peak at ∼21,100 cm^–1^, that is, 475 nm, and the gradual transition of the fluorescence peak toward the red on a nanosecond time scale.

In the sample containing glucose (red spectra), TRES data show a similar transition; however, the emission peak at 475 nm is already apparent at day 0, which may suggest that glucose accelerates the formation of this fluorescent residue. The direct inspection of TRES suggests at least two fluorescent residues.

**Figure 7:**
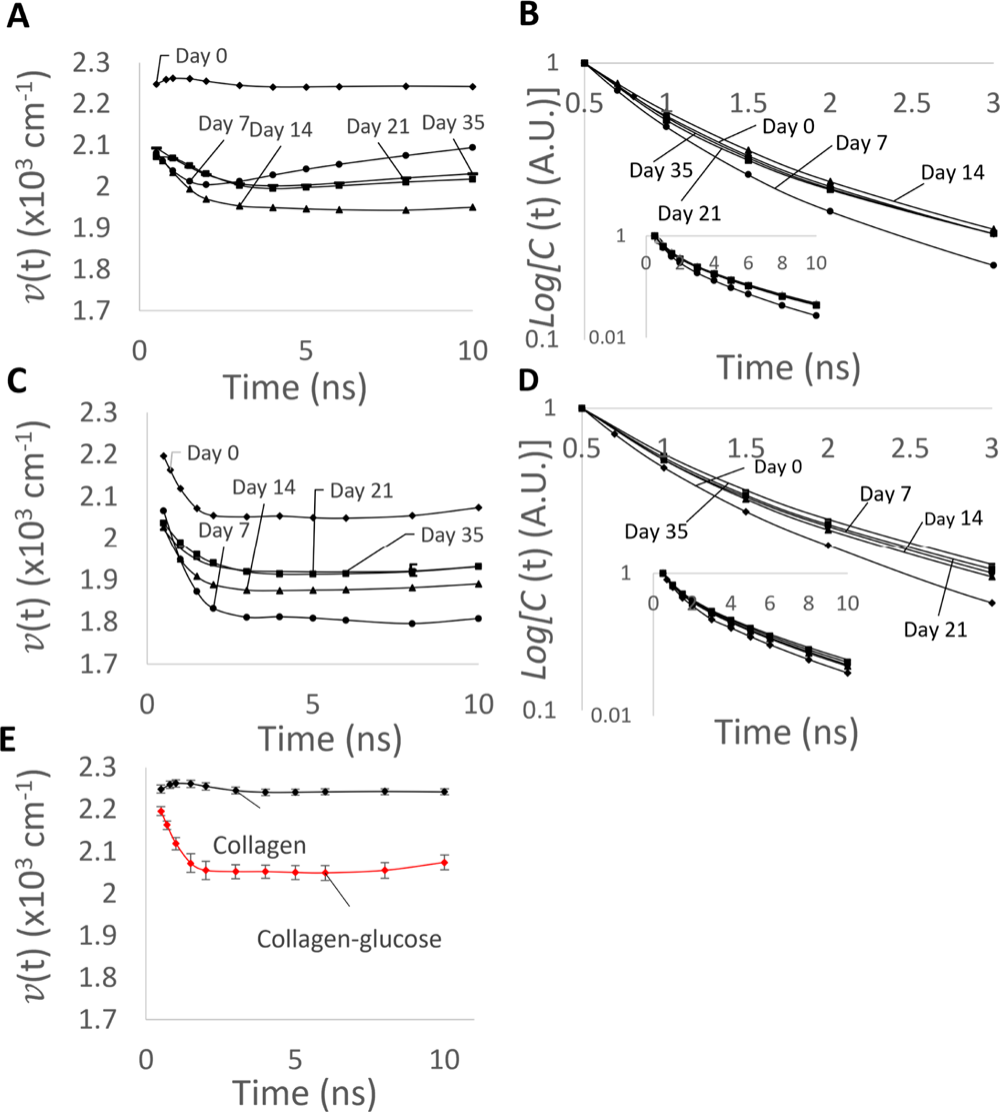
Plots (A,C) show the time evolution of the peak position *v*(*t*) of the emitting fluorophores for collagen and collagen–glucose, respectively, excited at 340 nm. The normalized time evolution of the fluorescence intensity C(*t*) is shown for collagen and collagen–glucose in plots (B,D). The inset shows the same data over a longer timer range. The five lines show day 0, day 7, day 14, day 21, and day 35. Part (E) shows (*t*) for collagen and collagen–glucose on day 0 for comparison, with the error bars showing the 90% confidence intervals for the fitted values.

Indeed, although the analysis of the Akaike information criterion (see Table T2) again requires the consideration of a one-component Toptygin model (*M* = 1), the analysis of the evolution of the *v*(*t*) parameter is characteristic for multicomponent kinetics (Figure 7), which we explain below.

The initial (Day 0) peak position of the free collagen spectrum starts at ∼22,500 cm^–1^ (Figure 7A) and at ∼22,000 cm^–1^ for collagen–glucose (Figure 7C) and they behave differently, see Figure 7E for comparison. There is no shift in *v*(*t*) following excitation for free collagen at day 0; however, the measurements taken on the later days show a substantial drop of the initial *v*(*t*) to ∼20,900 cm^–1^. All *v*(*t*) dependencies show a further red shift during the first 2 ns, and then the *v*(*t*) plots start to increase (which is most visible for the data obtained on day 7). Again, this observation contradicts the homogeneous dielectric relaxation process, where the monotonic red shift is expected. It can only be explained by the fluorescence of a number of species of individual fluorescence lifetimes, dielectric relaxation rates, and so forth. Obtaining *M* = 1 in the Akaike analysis can be again explained by the presence of aggregates characterized by a distribution of fluorescence characteristics, rather than a small number of aggregates of distinctively different parameters.

The *v*(*t*) for the collagen–glucose sample on day 0 drops from 22,000 to ∼20,500 cm^–1^ during the first 2 ns. In the case of older samples, the starting value of *v*(*t*) is ∼20,600 cm^–1^ which is also followed by a similar red shift within the first 2 ns. This behavior of *v*(*t*) demonstrates that in the presence of glucose, the formation of cross-links occurs differently, leading to only one type of the fluorescent cross-link.

The log(*C*(*t*)) curves (Figure 7B,D) seem to confirm this observation. The changes in the rates of the decays on different days in the pure collagen sample (Figure 7B) are not monotonic. Indeed, the decay in Day 7 is faster than in Day 0, and then it slows down again at the later days. This suggests the presence of multiple fluorescent cross-links whose contributions change over time. The decays observed in the glycated sample (Figure 7D), however, show a stable trend of decreasing decay rates, which is likely to be observed when there is a single or a set of uniform fluorescent residues.

To conclude, in a pure collagen sample, more than one type of fluorescent cross-link is formed in the 35 day period, while in the collagen–glucose sample, a single fluorescent form is created, demonstrating a substantial impact of glycation on collagen aggregation.

## Conclusions

The effects of glucose on collagen’s intrinsic fluorescence has been studied using a TRES-based technique. Our aim was to identify the characteristic features of the excited-state kinetics occurring in the glucose-triggered aggregation of collagen. Intrinsic fluorescence responses were collected for the excitation wavelengths 280 and 340 nm to observe changes in the fluorescence of intrinsic tyrosine and fluorescent cross-links.

It has been found that glucose does not have a substantial impact on the fluorescence of intrinsic Tyr (excitation at 280 nm). Indeed, as glucose binds to collagen at lysine residues, and lysine makes up only 3.9% of collagen,^15,35^ formation of lysine–glucose complexes is not likely to impact Tyr’s local microenvironment. Nevertheless, the TRES of both free collagen and collagen–glucose samples excited at 280 nm do change over time, which is due to protein aggregation.

Glucose, however, does have a substantial influence on the formation of pepsin-digestible collagen cross-links emitting at λ > 400 nm (excitation at 340 nm). The observed changes in TRES show the impact of glucose on the formation pathways of new cross-links and protein aggregates.

However, as the TRES approach that we have used does not assume any specific model of the underlying excited state kinetics; further studies will be required to identify the best fit model of the kinetics and for determining the molecular mechanisms involved.

## Supporting Information Available

Examples of raw TCSPC data analysis, calculated TRES values versus.TRES model curves, AIC_c_ definition, and examples of calculated values (PDF)
