# Supplementary material for: Collagen Glycation Detected by Its Intrinsic Fluorescence
Source: J Phys Chem B. 2021 Sep 24;125(39):11058–66. doi: 10.1021/acs.jpcb.1c05001 (PMC8793138; doi:10.1021/acs.jpcb.1c05001)
Supplement: Supplementary file 1 — jp1c05001_si_001.pdf [file jp1c05001_si_001.pdf]

Collagen Glycation Detected by its Intrinsic Fluorescence

Supporting Information

Rhona Muir[a], Shareen Forbes[b], David J.S. Birch[a], Vladislav Vyshemirsky[c] and Olaf J. Rolinski*[a]

[a]Photophysics Group, Department of Physics, University of Strathclyde, Scottish Universities Physics Alliance, Glasgow G4 0NG, UK.

[b] BHF Centre for Cardiovascular Science, The Queen's Medical Research Institute, University of Edinburgh, Edinburgh EH16 4TJ, UK.

[c]School of Mathematics and Statistics, University of Glasgow, Glasgow G12 8QQ, UK.

The supporting information file includes

1. Raw TCSPC data analysis.
2. Calculated TRES values vs. TRES model (Eqtn 5) curves.
3. Akaike's Information Criterion (AICc), definition used and the results for the collagen and collagen-glucose samples.

1. Raw TCSPC data analysis

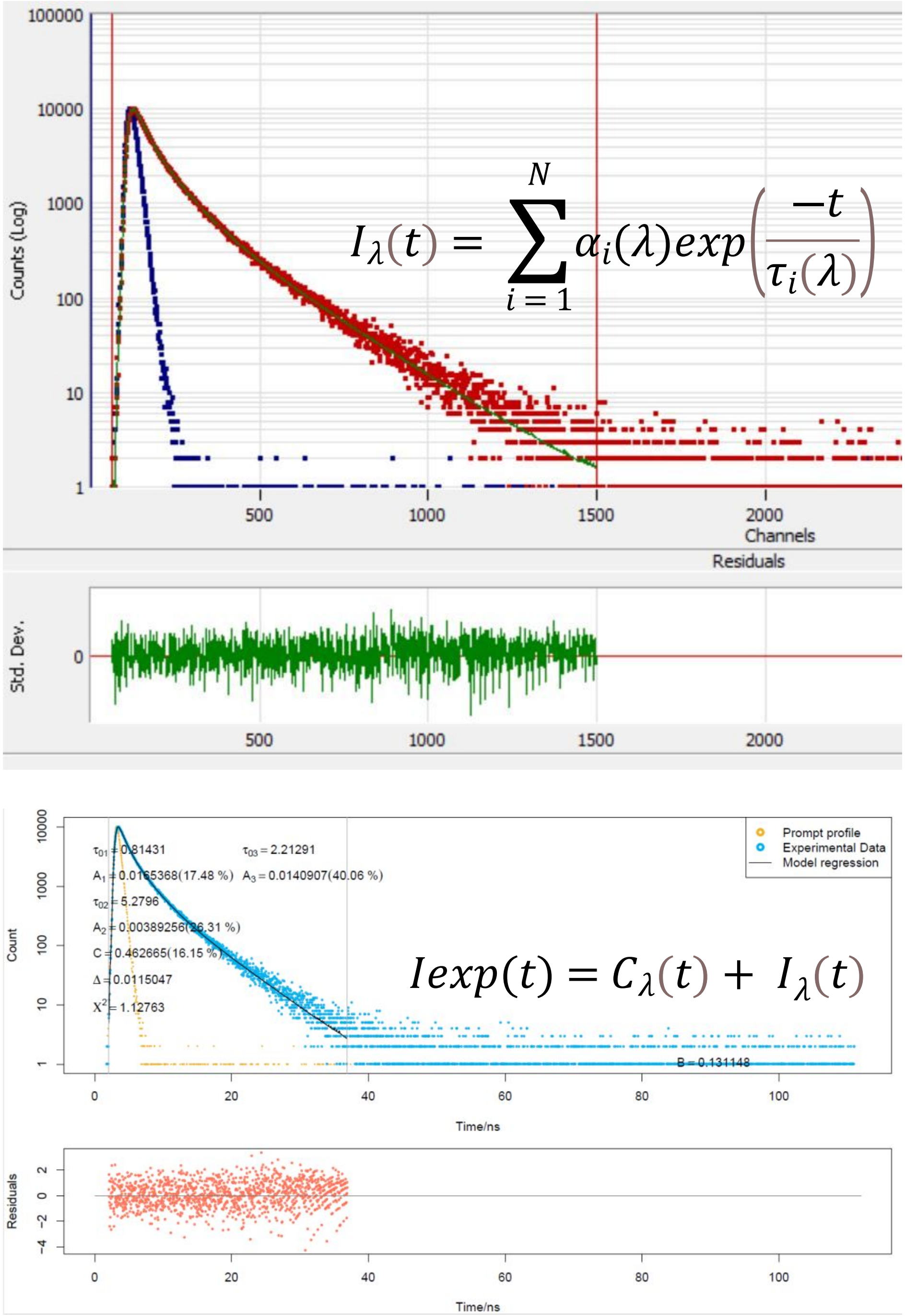

	τ_1(ns)	A1 /%	τ_2(ns)	A2 /%	τ_3(ns)	A3 /%	τ_4(ps)	A4 /%	C (%)	X^2
3+1 - exponential	0.84	0.0173 (13.63)	2.25	0.0139 (29.41)	5.30	0.00382 (19.02)	13.67	2.96 (37.94)		1.10
3 - exponential + scatter function	0.81	0.0165 (17.48)	2.22	0.0141 (40.06)	5.28	0.00389 (26.31)			16.15	1.13

Figure S1. Result of fitting of the fluorescence intensity decay of the sample collagen-glucose, day 0. Excitation wavelength 280 nm, detection wavelength 330 nm.

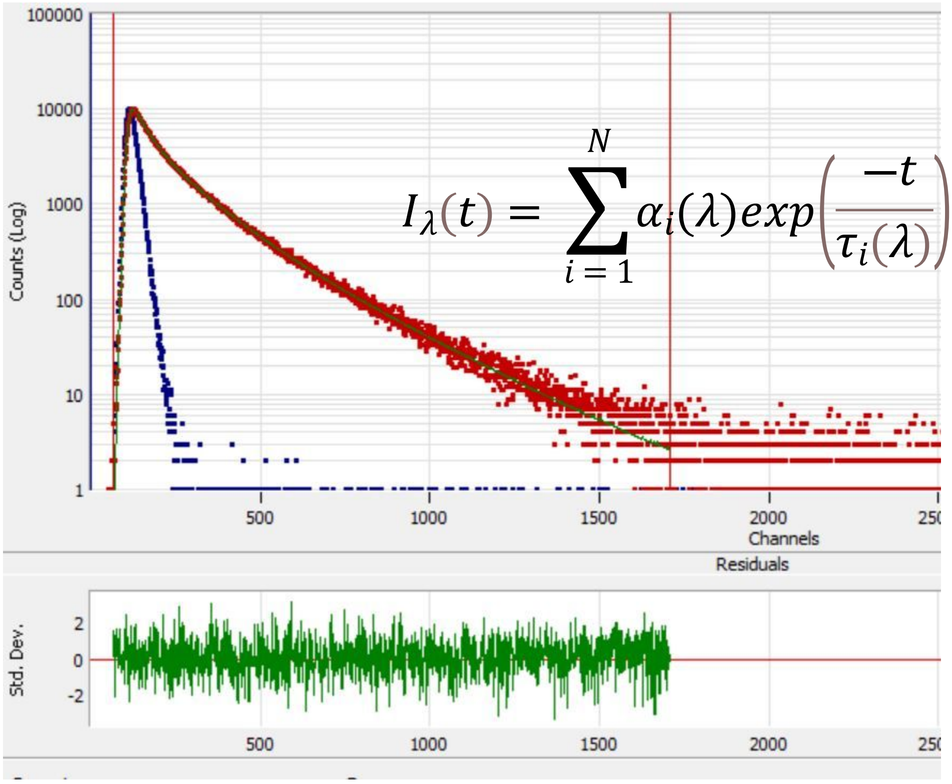

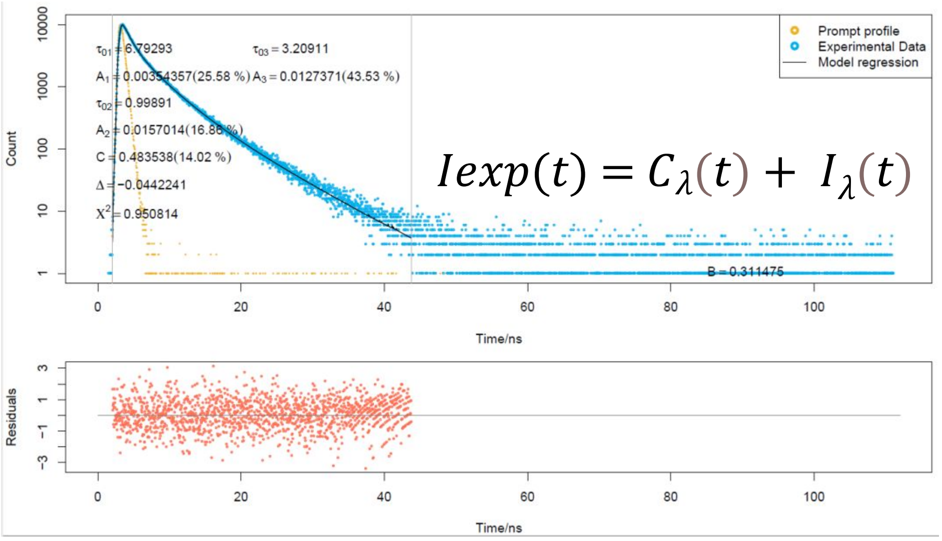

	τ_1(ns)	A1 /%	τ_2(ns)	A2 /%	τ_3(ns)	A3 /%	τ_4(ps)	A4 /%	C (%)	X^2
3+1 - exponential	0.99	0.0159 (12.7)	3.13	0.0128 (32.54)	6.65	0.00387 (20.82)	13.67	3.07 (33.93)		0.97
3 - exponential + scatter function	0.99	0.0157 (16.86)	3.21	0.0127 (43.53)	6.79	0.00354 (25.58)			14.02	0.95

Figure S2. Result of fitting of the fluorescence intensity decay of the sample free collagen, day 14. Excitation wavelength 280 nm, detection wavelength 360 nm.

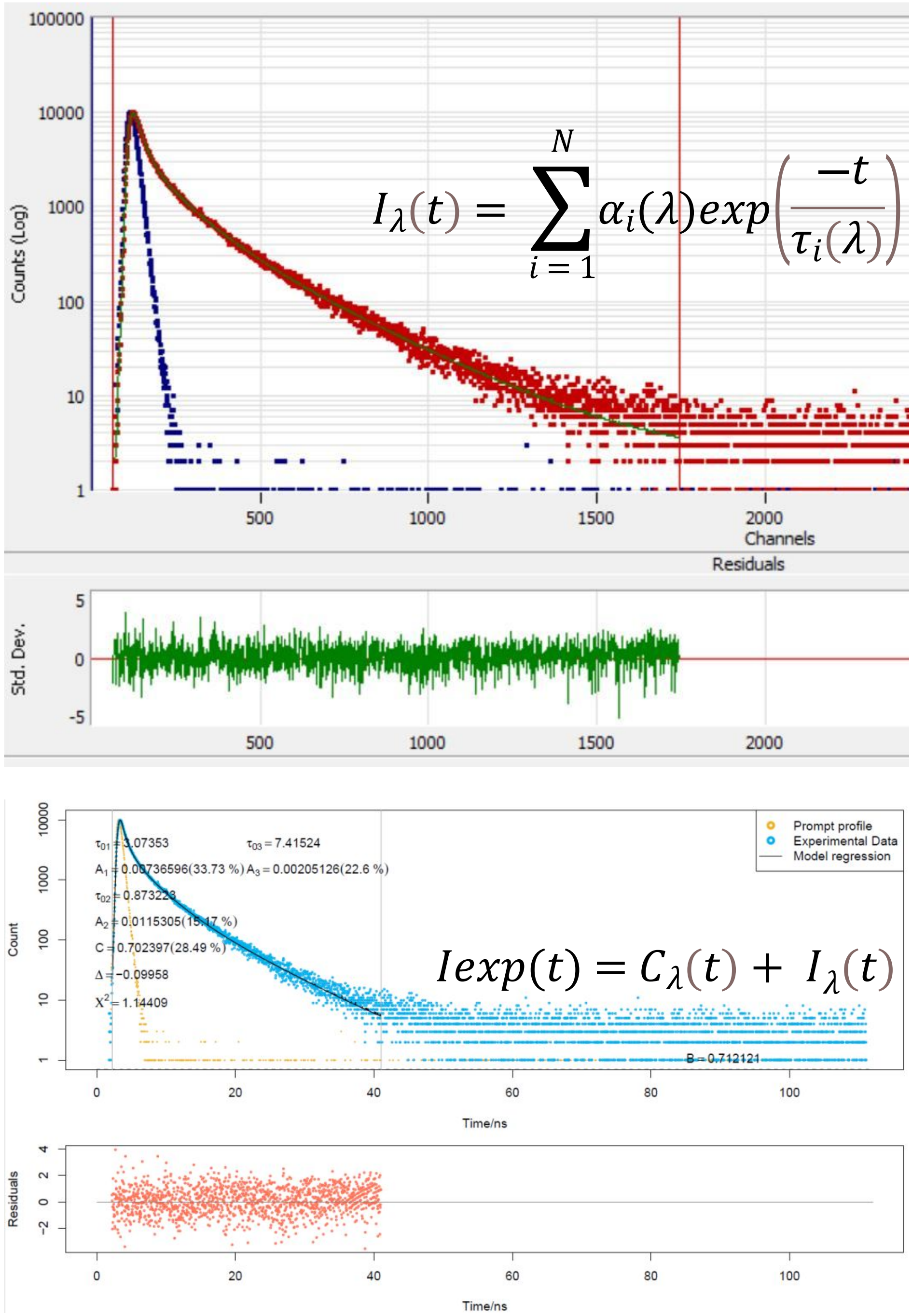

	τ_1(ns)	A1 /%	τ_2(ns)	A2 /%	τ_3(ns)	A3 /%	τ_4(ps)	A4 /%	C (%)	X^2
3+1 - exponential	0.90	0.0118 (9.65)	3.10	0.00727 (20.55)	7.34	0.00207 (13.86)	13.67	4.49 (55.94)		1.15
3 - exponential + scatter function	0.87	0.0115 (15.17)	3.07	0.00767 (33.73)	7.42	0.00205 (22.16)			28.49	1.14

Figure S3. Result of fitting of the fluorescence intensity decay of the sample collagen-glucose, day 28. Excitation wavelength 280 nm, detection wavelength 390 nm.

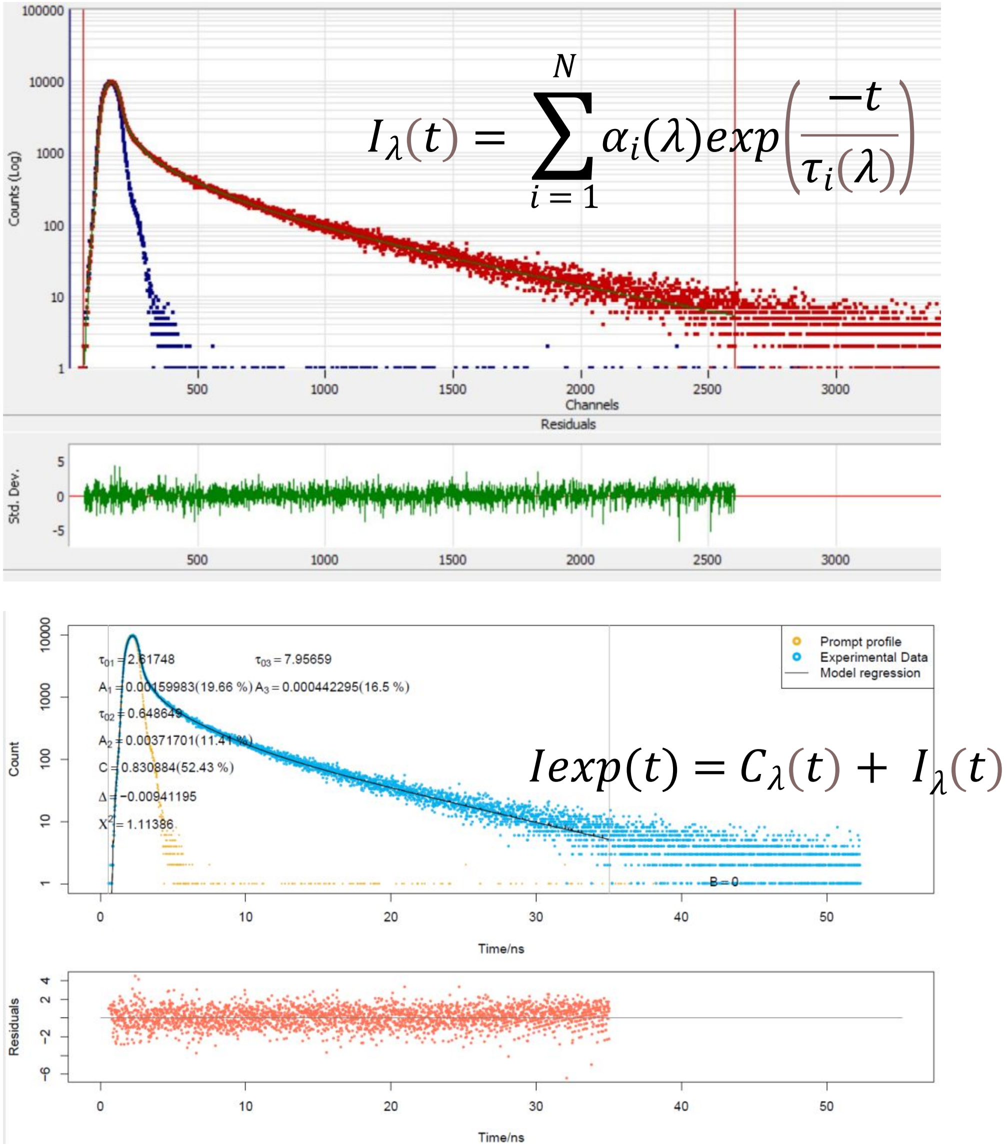

	τ_1(ns)	A1 /%	τ_2(ns)	A2 /%	τ_3(ns)	A3 /%	τ_4(ps)	A4 /%	C (%)	X^2
3+1 - exponential	0.63	0.00367 (5.06)	2.44	0.00164 (8.71)	7.43	0.000512 (8.28)	6.74	5.32 (77.95)		1.11
3 - exponential + scatter function	0.65	0.00372 (11.41)	2.62	0.00160 (19.66)	7.96	0.000442 (16.5)			52.43	1.11

Figure S4. Result of fitting of the fluorescence intensity decay of the sample free collagen, day 0. Excitation wavelength 340 nm, detection wavelength 440 nm.

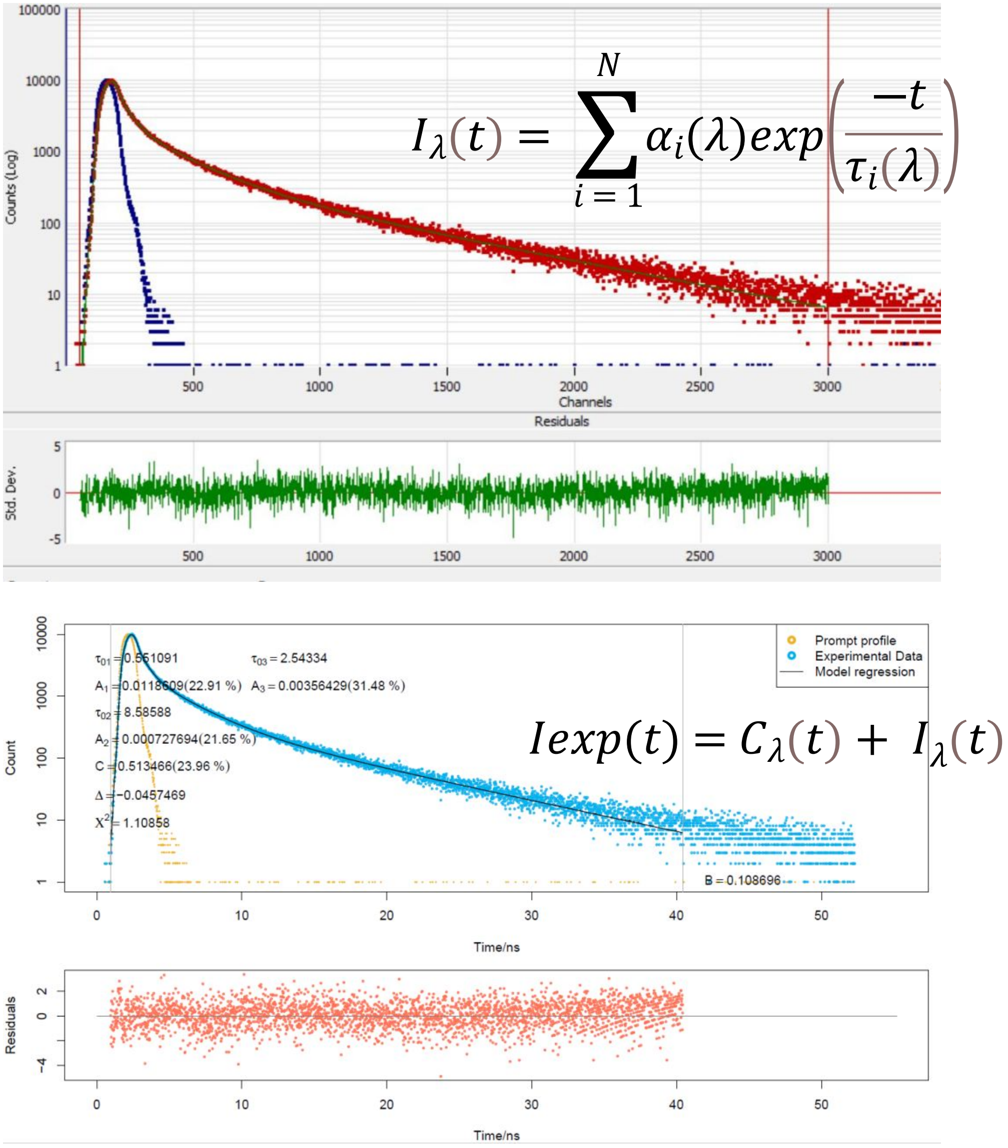

	τ_1(ns)	A1 /%	τ_2(ns)	A2 /%	τ_3(ns)	A3 /%	τ_4(ps)	A4 /%	C (%)	X^2
3+1 - exponential	0.52	0.0132 (15. 8)	2.46	0.00371 (21.04)	8.34	0.000778 (14.97)	6.74	3.10 (48.19)		1.11
3 - exponential + scatter function	0.56	0.0119 (22.91)	2.54	0.00356 (31.48)	8.59	0.000728 (21.65)			23.96	1.11

Figure S5. Result of fitting of the fluorescence intensity decay of the sample collagen-glucose, day 14. Excitation wavelength 340 nm, detection wavelength 400 nm.

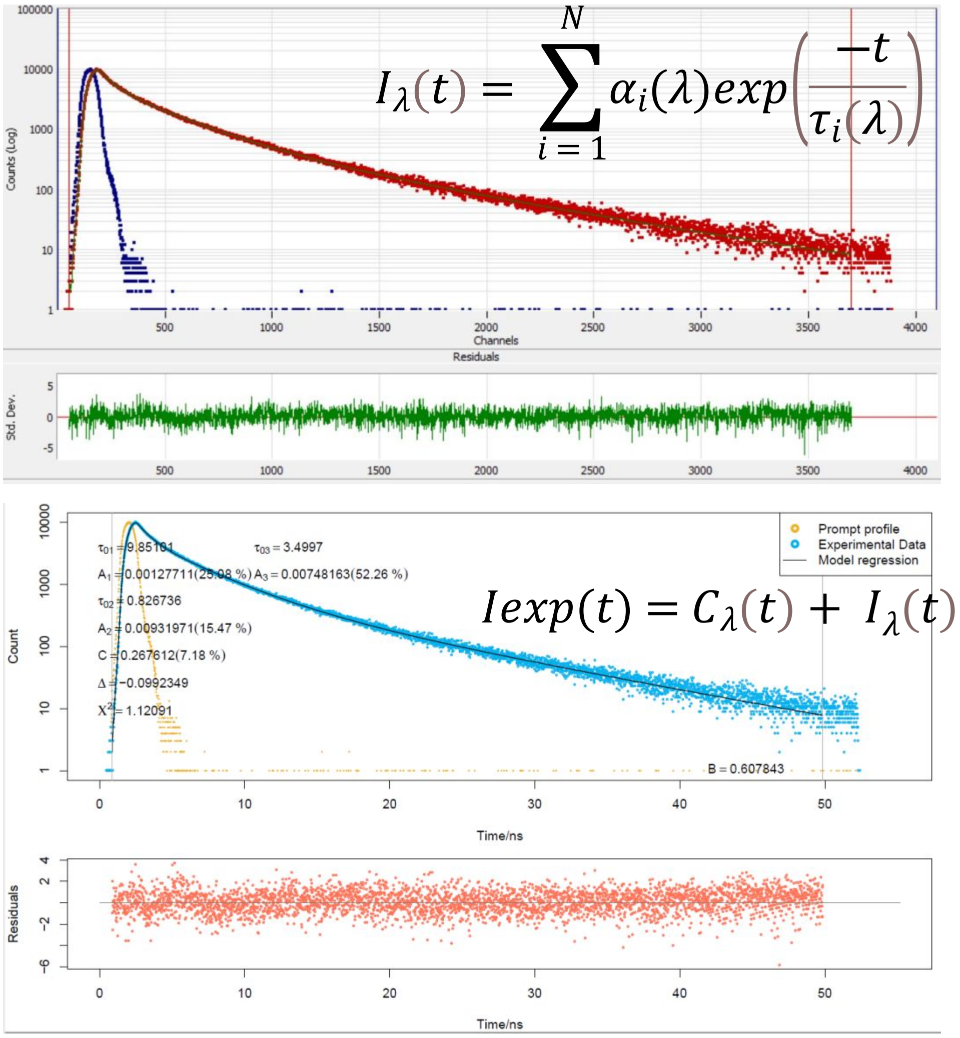

	τ_1(ns)	A1 /%	τ_2(ns)	A2 /%	τ_3(ns)	A3 /%	τ_4(ps)	A4 /%	C (%)	X^2
3+1 - exponential	0.81	0.00932 (12.94)	3.39	0.00755 (44.04)	9.39	0.00144 (23.23)	6.74	1.71 (19.79)		1.09
3 - exponential + scatter function	0.83	0.00932 (15.47)	3.50	0.00748 (52.26)	9.85	0.00128 (25.08)			7.18	1.12

Figure S6. Result of fitting of the fluorescence intensity decay of the sample collagen-glucose, day 35. Excitation wavelength 340nm, detection wavelength 480 nm.

2. Calculated TRES values vs. TRES model (Eqtn 5) curves.

A

B

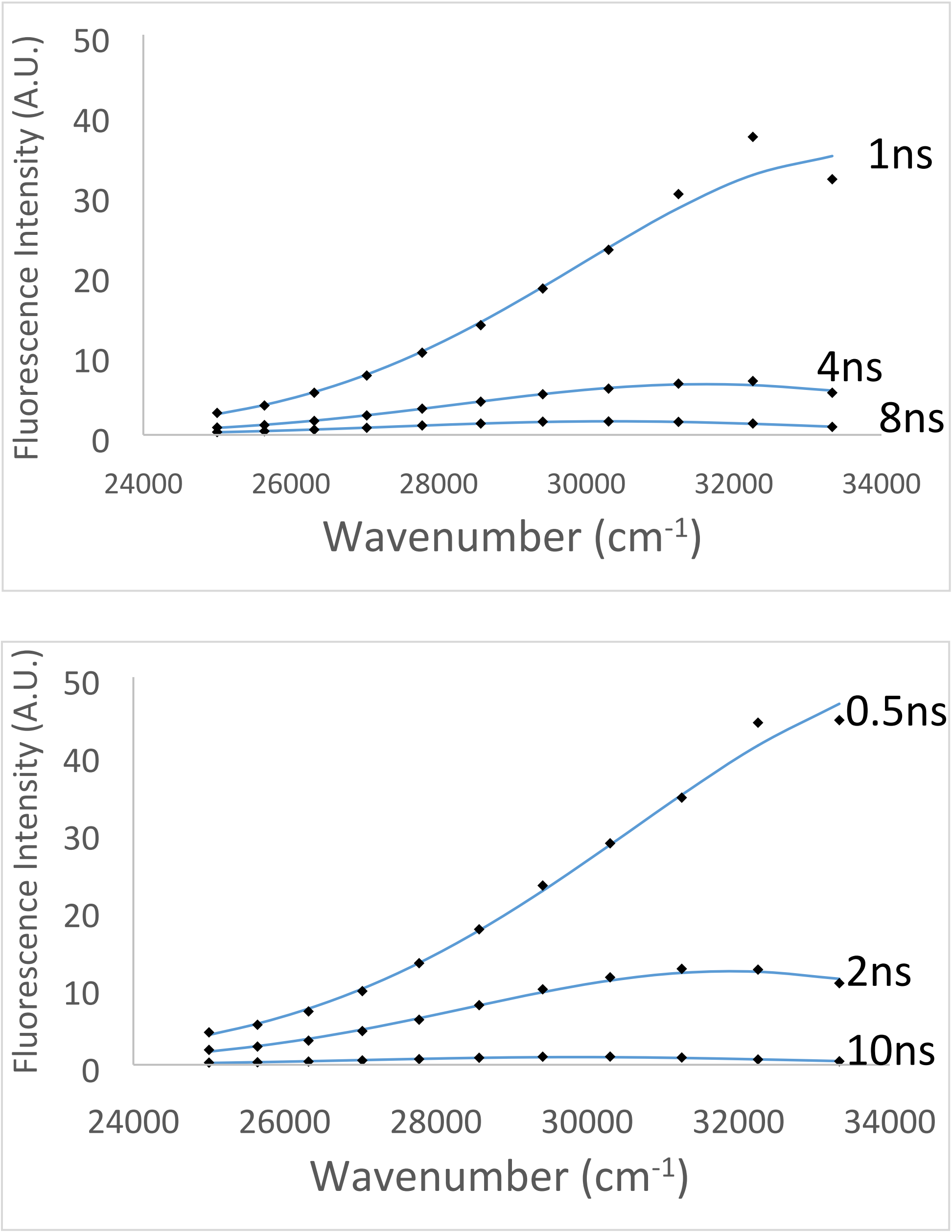

Figure S7. Examples of the model and experimental TRES for excitation 280 nm. TRES calculated from experimental data are shown as black markers, while the model is shown as a blue line. Part A shows an example from collagen-glucose at day 0, while part B shows an example from collagen at day 56.

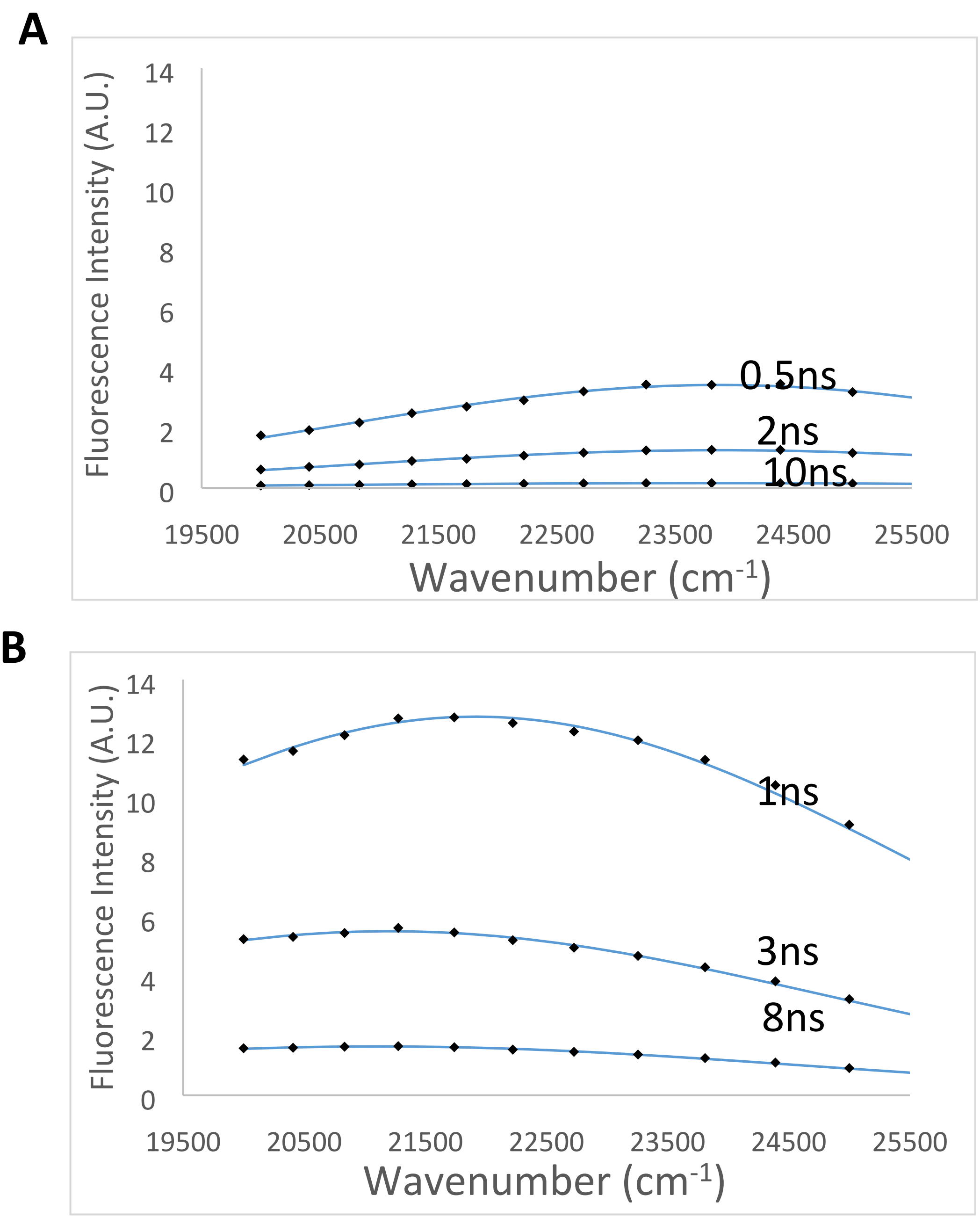

Figure S8. Examples of the model and experimental TRES for excitation 340 nm. TRES calculated from experimental data are shown as black markers, while the model is shown as a blue line. Part A shows an example from collagen at day 0, while part B shows an example from collagen-glucose at day 21.

3. Akaike's Information Criterion (AIC_c)

AIC measures the appropriateness of the model based on the residual sum of squares RSS from the nonlinear least squares fitting, the number of parameters k, and the number of data points n. Since the sample size is small an additional correction factor was used, giving the corrected Akaike's Information Criterion (AIC_c)

$$AIC_c = nlog\left(\frac{RSS}{n}\right) + nlog(2\pi) + n + 2k + \frac{2k(k+1)}{n-k-1}$$

Since n is the same in all our cases, the constant terms can be dropped giving

$$AIC_c = nlog\left(\frac{RSS}{n}\right) + n + 2k + \frac{2k(k+1)}{n-k-1}$$

A smaller AIC_c indicates a more parsimonious model i.e. the simpler model with the least assumptions and variables, but the greatest explanatory power. (Burnham KP and Anderson DR, Model Selection and Multimodel Inference, A Practical Information-Theoretic Approach, Springer 2002.)

TRES Spectra		AIC_c	
		Single Toptygin model	Double Toptygin model
Collagen Day 0	1ns	49.25	47.67
	4ns	7.14	9.15
	8ns	-34.57	-27.59
Collagen –Glucose Day 21	1ns	40.24	33.59
	4ns	-4.86	-4.31
	8ns	-42.71	-36.27
Collagen Day 56	1ns	40.96	42.72
	4ns	-3.61	6.32
	8ns	-40.68	-28.10

Table T1. Examples of AICc values for the one-and two-component Toptygin model of the TRES for free collagen and collagen glucose when exciting at 280 nm. The smaller AICc value indicates a more adequate model. A small selection of TRES spectra at different days and times after excitation have been included.

TRES Spectra		AICc	
		Single Toptygin model	Double Toptygin model
Collagen Day 0	1ns	-71.71	-68.21
	4ns	-108.57	-101.68
	8ns	-132.53	-119.44
Collagen –Glucose Day 21	1ns	-32.26	-28.97
	4ns	-59.25	-50.79
	8ns	-81.12	-82.58
Collagen Day 56	1ns	-53.64	-46.18
	4ns	-86.15	-79.81
	8ns	-106.51	-96.47

Table T2. Examples of AICc values for the one-and two-component Toptygin model of the TRES for free collagen and collagen glucose when exciting at 340 nm. The smaller AICc value indicates a more adequate model. A small selection of TRES spectra at different days and times after excitation have been included**.**
